# Seroprevalence of *Triatoma virus* (*Dicistroviridae*: *Cripaviridae*) antibodies in Chagas disease patients

**DOI:** 10.1186/s13071-015-0632-9

**Published:** 2015-01-17

**Authors:** Jailson FB Querido, María G Echeverría, Gerardo A Marti, Rita Medina Costa, María L Susevich, Jorge E Rabinovich, Aydee Copa, Nair A Montaño, Lineth Garcia, Marisol Cordova, Faustino Torrico, Rubén Sánchez-Eugenia, Lissete Sánchez-Magraner, Xabier Muñiz-Trabudua, Ibai López-Marijuan, Gabriela S Rozas-Dennis, Patricio Diosque, Ana M de Castro, Carlos Robello, Julio S Rodríguez, Jaime Altcheh, Paz M Salazar-Schettino, Marta I Bucio, Bertha Espinoza, Diego MA Guérin, Marcelo Sousa Silva

**Affiliations:** Centre for Malaria and Tropical Diseases, Instituto de Higiene e Medicina Tropical, Universidade Nova de Lisboa, Lisbon, Portugal; Cátedra de Virología, Facultad de Ciencias Veterinarias Universidad Nacional de La Plata (UNLP-CONICET), La Plata, Argentina; Centro de Estudios Parasitológicos y de Vectores (CEPAVE-CCT- La Plata -CONICET - UNLP), La Plata, Argentina; Laboratorio de Biología Molecular IIBISMED, Facultad de Medicina, Universidad Mayor de San Simón, Cochabamba, Bolivia; Facultad de Medicina, Universidad Mayor de San Simón, Cochabamba, Bolivia; Unidad de Biofísica (UBF, CSIC, UPV-EHU), Leioa, Bizkaia Spain; Departamento de Biología, Bioquímica y Farmacia, and Grupo Biofísica, Departamento de Física, Universidad Nacional del Sur, Bahía Blanca, Argentina; Unidad de Epidemiología Molecular del Instituto de Patología Experimental, Facultad de Ciencias de la Salud, Universidad Nacional de Salta, Salta, Argentina; Instituto de Patologia Tropical e Saúde Pública, Universidade Federal de Goiás, Goiania, Brazil; Unidad de Biología Molecular Instituto Pasteur de Montevideo, Mataojo 2020, CP11400 Montevideo, Uruguay; CENPALAB, Delegación CIMTA, Santiago de Cuba, Cuba; Parasitologia-Chagas, Hospital de Niños R. Gutierrez, Buenos Aires, Argentina; Departamento de Microbiología y Parasitología, Facultad de Medicina (LBP-DMP-FM), Universidad Nacional Autónoma de México, Mexico City, DF México; Departamento de Inmunología, Instituto de Investigaciones Biomédicas, Universidad Autónoma de México, Mexico City, DF México; Departamento de Bioquímica y Biología Molecular, Universidad del País Vasco (EHU/UPV), Bizkaia, Spain; Programa de Pós-graduação em Bioquímica, Departamento Bioquímica, Universidade Federal do Rio Grande do Norte, Natal, Brazil

**Keywords:** *Triatoma virus*, *Dicistroviridae*, *Trypanosoma cruzi*, Triatomines, Chagas disease, Mathematical model, Biological control, Passive viral exposure

## Abstract

**Background:**

Chagas disease is caused by *Trypanosoma cruzi*, and humans acquire the parasite by exposure to contaminated feces from hematophagous insect vectors known as triatomines. Triatoma virus (TrV) is the sole viral pathogen of triatomines, and is transmitted among insects through the fecal-oral route and, as it happens with T. cruzi, the infected insects release the virus when defecating during or after blood uptake.

**Methods:**

In this work, we analysed the occurrence of anti-TrV antibodies in human sera from Chagas disease endemic and non-endemic countries, and developed a mathematical model to estimate the transmission probability of TrV from insects to man, which ranged between 0.00053 and 0.0015.

**Results:**

Our results confirm that people with Chagas disease living in Bolivia, Argentina and Mexico have been exposed to TrV, and that TrV is unable to replicate in human hosts.

**Conclusions:**

We presented the first experimental evidence of antibodies against TrV structural proteins in human sera.

**Electronic supplementary material:**

The online version of this article (doi:10.1186/s13071-015-0632-9) contains supplementary material, which is available to authorized users.

## Background

Chagas disease is an arthropod-borne zoonotic disease also known as American trypanosomiasis, and caused by the protozoan flagellate parasite *Trypanosoma cruzi* [1]. This disease is endemic in most of the Latin American countries, and between 7 to 8 million have been estimated to be infected people in the American continent [2]. *T. cruzi* is transmitted to humans and to wild and domestic animals by many species of haematophagous insects known as triatomines (*Hemiptera*: *Reduviidae*) [1]. The most common source of infection is when the parasite reaches the bloodstream of the bitten person who scratches the bite site and contaminates the skin wound with the insect’s feces containing the parasites. The parasite can also infect the host through the surfaces of mucosa and/or conjunctiva. In contrast to salivarian pathogens such as *Plasmodium* spp., *Leishmania* spp., and dengue virus, in which transmission to humans occurs with 1 to 4 insect bites, the probability of being infected with *T. cruzi,* a stercorarian parasite, is much smaller: between 900 and 4,000 bites by an infected triatomine are needed for *T. cruzi* infection to occur [3,4].

*Triatoma virus* (TrV) *(Dicistroviridae: Cripavirus)* is a non-enveloped virus [5] and its genome contains two open reading frames (ORFs). ORF1 (5,387 nt in length) is responsible for encoding non-structural proteins such as RNA helicase, cysteine protease (3C) and RNA-dependent RNA polymerase (RdRp), while ORF2 (2,606 nt in length) encodes the structural proteins VP1, VP2, VP3, and the minor protein VP4 [6]. The precursor protein of VP2 and VP4 is known as VP0. This virus was first isolated from *Triatoma infestans* [7,8], the main vector of Chagas in Argentina and neighbouring countries. The area of Argentina in which TrV was found covers provinces along the Andes, the central part of the country and also the region known as Gran Chaco; the latter is a vast flat area (approximately one million km^2^) that extends through northern Argentina, south-eastern Bolivia, north-western Paraguay, and a small area of south-western Brazil.

The virus replicates in the cells of the intestinal epithelium of triatomines, and the viral infection causes leg paralysis, delayed development, reduced fertility, and mortality [7-9]. Previous studies described the mechanisms of TrV transmission among triatomines and found that it can occur through the fecal-oral route, cannibalism, or transovarian infection [10]. In addition to *T. infestans*, others species of triatomines from wild areas in Argentina [11] and from an insectary in Brazil were found to be infected with TrV [12]. This suggests that TrV can be distributed to other areas of the American continent.

To date, TrV is the only known viral pathogen of triatomines^a^. Recent studies have established TrV prevalence in sylvatic populations of triatomines in the north-western provinces of Argentina and determined that it can reach up to 20% [13]. The virus infects several triatomine species living in allecotopes [11]. One of the salient results of an extensive study by Marti and colleagues was that the average prevalence of TrV in *T. infestans* was approximately 9.7% [13], many times greater than the average prevalence of other parasites, pathogens, and symbionts found in the same sample of triatomines, and in particular of *T. cruzi*, which was found in approximately 1.3% of the examined specimens.

It has been considered that millions of people, as well as wild and domestic animals living in Chagas disease endemic areas, may have been in contact with TrV [10]. This assumption has been supported by the observation that hens used to feed triatomines infected with TrV, had positive serology for anti-TrV antibodies [10], suggesting that, similarly to what happens with *T. cruzi*, humans or animals can be inoculated with TrV particles existing in the feces of triatomines.

In this study, we searched for anti-TrV antibodies in human sera of people living in both countries where Chagas disease is endemic and non-endemic. Also, we developed a mathematical model to estimate human exposure to TrV, and compared the transmission rate of TrV relative to that of *T. cruzi*.

## Methods

### Study population

In this work, we searched for anti-TrV antibodies in 410 human sera samples from four endemic and two non-endemic Chagas disease countries (Table 1). We used 117 sera samples from Bolivia, 100 samples from Mexico, 122 samples from Argentina, 50 samples from Brazil, 10 samples from Cuba and 11 samples from Portugal. Aside from the samples used as controls, 62% of the sera samples came from patients with Chagas disease. This was to guarantee that most of the patients had been in contact with triatomines and consequently had an increased likelihood of exposure to TrV.

**Table 1:** **Positive and negative sera for** ***Triatoma virus*** **by country of origin resulting from different types of analysis**

**Country**	**Sera analysed**	**Chagas patients (%)**	**ELISA 1 positive**	**ELISA 2 negative**	**WB positive**	**RT-PCR negative**	**Negative control**
Argentina	122	40	41	50	36	50	12
Bolivia	117	68	11	-	-	-	20
Brazil	50	100	0	-	-	-	22
Cuba	10	0*	0	-	-	-	5
Mexico	100	70	9	-	-	-	8
Portugal	11	100^#^	0	-	-	-	8
Total	**410**	**63**	**61**	**50**	**36**	**50**	**81**

Column 1 identifies the country of origin for the samples; results of the TrV sera analyses. Numbers in columns 4**–**7 indicate the number of serum samples employed in each analysis. ELISA assay 1 corresponds to reactivity against structural proteins, and ELISA assay 2 corresponds to reactivity against the non-structural protein RdRp. WB refers to immunoblotting analysis against the structural proteins of TrV. RT-PCR refers to reverse transcriptase-PCR*. Cuba is a non-endemic country for Chagas disease. ^#^The sera from Portugal are imported case of Chagas disease, mainly from Brazil.

In general, for each country and for all serological assays, we analysed, as negative controls, sera from healthy people with the same region. In particular, for the controls of Argentina, healthy people sera came from a TrV-free province. In the cases of Cuba and Portugal (the non-endemic Chagas disease countries), we used sera from people who had never been in countries endemic for Chagas disease.

### Ethics statement

Sera samples were obtained and analyzed in accordance with the institutional Ethics Committees regulations from the countries where they were analysed. Authorizations of ethics committees were obtained individually in each country presented in the study: Mexico (Faculty of Medicine – *Universidad Autonoma de Mexico* - FMED/CI/RGG/024/08/2006 project # 035–2009); Argentina (Institutional Bioethics Committee – Bahia Blanca – Doc # 017/2010 from 17 October 2013); Portugal (Institutional Bioethics Committee at *Instituto de Higiene e Medicina Tropical – Universidade Nova de Lisboa*); Brazil (Institutional Bioethics Committee at *Hospital São Salvador* – Goiânia – GO); Bolivia (Institutional Bioethics Committee at Faculty of Medicine – *Universidad Mayor de San Simon*); Cuba (consent of the ethics and biosafety committee at CENPALAB).

### ELISA specific for the detection of antibodies anti-*Triatoma virus* structural proteins

Natural empty TrV particles (e-TrV) purified from the feces of infected insects [14] were used as antigens. For the total anti-TrV IgG in human sera determination, e-TrV particles (100 ng/well) were diluted in 0.1 M bicarbonate buffer (pH 8.5) and incubated overnight at 4°C in 96-well micro-plates (Nunc, Denmark). In this indirect enzyme-linked immunosorbent assay (ELISA), we used the human sera at a dilution of 1:10,000. Anti-human IgG (whole molecule) − HRP antibody produced in rabbit (1:4000, Sigma-Aldrich, USA) was used as a secondary antibody and bound antibody was detected by incubation with *o*-Phenylenediaminedihydrochloride (Sigma-Aldrich, USA) and water (Sigma-Aldrich, USA) for 30 min. The reaction was stopped with 2MH_2_SO_4_, and then the absorbance or Optical Density (OD) was read at 490 nm (Bio-Rad Microplate Reader 680 XR, USA). Data from the ELISA assays were processed and analysed by GraphPad Prism 6® (GraphPad Software; USA).

### ELISA specific for the detection of antibodies anti-*Triatoma virus* non-structural protein (RdRp)

We used the non-structural protein RdRp from TrV as antigen. The protein was obtained by recombinant expression in *E. coli* (see below). The procedure was the same as described for ELISA assay 1, except that we used an antibody dilution of 1:5,000. Each serum sample was diluted in the antibody buffer and incubated overnight with 6 μg of total *E. coli* proteins. This reduced the background due to the occurrence of natural anti-*E. coli* antibodies in human sera.

In both ELISAs, the cut-off values were obtained by using negative control samples (see study population above), by establishing the cut-off value as the mean of the ODs plus three times the standard deviation, which allowed us to discriminate between the negative and positive sera.

### Molecular identification of *Triatoma virus* by Reverse Transcriptase-PCR (RT-PCR)

For molecular identification of TrV, we performed RNA extraction and purification using TRIzol® Reagent (Life Technologies^TM^, USA) of all positive sera (n = 41) and some indeterminate sera (n = 9) from Argentina (previously analysed by ELISA assay 1). This subgroup of sera will be referred hereafter as SeraA^+^. As a positive control, we used RNA extracted from TrV that was diluted in sera from Portuguese patients who had never been in Chagas disease endemic areas.

The RT-PCR was performed according to the literature [15,16] using the following primer pair: TrV sense −5′ TCAAAACTAACTATCATTCTGG 3′(nt 7427 to 7448 in TrV ORF2 sequence) and TrV anti-sense - 5′TTCAGCCTTATTCCCCCCC ′3 (nt 8240–8258), with an expected product of 832 bp.

### Expression and purification of the recombinant *Triatoma virus* non-structural protein RdRp

ORF1 encodes for the non-structural protein precursor NS, which contains the functional proteins helicase, protease, and RdRp. The cDNA fragment corresponding to a domain containing a piece of RdRp (nucleotides 4275–5936) was obtained by RT-PCR using the following primers: sense 5′-CATG*CCATGG*GAGGAGTTAGTCAACTACC-3′ (NcoI restriction site in italics) and antisense 5′-CCG*CTCGAG*CATAGTCAAGTCCGTAGGATTCC-3′ (XhoI restriction site in italics). The cDNA was then inserted into a pET-28a vector (Novagen, Germany). Bacteria were grown in Luria-Bertani liquid medium with 0.025 mg/ml kanamycin at 37°C to A_600_ = 0.6-0.8. Then, 1 mM IPTG was added for induction, and expression proceeded for 3 h at 37°C. Cells were harvested by centrifugation (6,000 g, 10 min, 4°C).

The cell pellets were resuspended in lysis buffer (500 mM NaCl, 20 mM Tris, pH 8.0) and were lysed by sonication (25 cycles, 10 s on + 20 s off) in a Soniprep 150 MSE probe sonicator. The suspensions were then centrifuged at 30,000 g for 20 min at 4°C, and the inclusion bodies, recovered in the pellet, were washed three times using wash buffer (2 M urea, 500 mM NaCl, 1% (v/v) Triton X-100, 20 mM Tris, pH 8.0). Finally, the pellets were washed twice with PBS to remove Triton X-100 and urea completely. To confirm protein expression, the samples were tested by SDS-PAGE and by anti-His tag Western blot.

To isolate the recombinant RdRp protein from the *E. coli* proteins, we performed protein purification from polyacrylamide gels by sonication extraction [17].

### Animal model mimicking *Triatoma virus* infection and polyclonal anti-*Triatoma virus* antibody production in mice

Sera of mice inoculated with TrV were used as a positive control for antigenic analyses. These positive sera were produced in female *Mus musculus* BALB/c mice between 5 and 8 weeks of age, obtained from the *Instituto de Higiene e Medicina Tropical*, Lisbon, Portugal. A group of five mice were inoculated (intraperitoneal injection) with 3 μg of TrV. Thirty days after inoculation, the animals were anaesthetized to proceed the blood collection through the cardiac puncture, and hyper-immune sera were obtained according to the procedure reported in our previous study [18].

A group of five mice were used to produce polyclonal anti-RdRp antibodies. The mice were inoculated with 100 μg of recombinant RdRp mixed with 100 μl of Freund’s complete adjuvant (Sigma-Aldrich, USA). Fifty days after the first inoculation, the animals were inoculated with 100 μg of recombinant RdRp mixed with 100 μl of Freund’s incomplete adjuvant (Sigma-Aldrich, USA), and ten days after the second inoculation, the animals were sacrificed and hyper-immune sera were obtained using the same procedure described in the previous paragraph.

### Ethics statement

Animal experimentation was performed in accordance with existing legislation in Portugal (*Decreto-Lei* number 113/2013) and in accordance with European directive (Directive - 2010/63/EU).

### Immunoblotting analysis

Empty-TrV particles (5 μg/well) were loaded onto 12% polyacrylamide SDS-PAGE gels after denaturation at 95°C for 5 min with β-mercaptoethanol. After running at 100 V, the gel was equilibrated in a transfer buffer (192 mM glycine, 20% methanol, 25 mM Tris pH 9.0) for 20 min. Proteins were transferred from the gel to nitrocellulose membranes using a semi-dry trans-blot system at 15 V for 40 min. After the transference, membranes were washed with PBS and blocked by incubation for 1 hour with 3% BSA in PBS. In the Western blot analyses, the sera were analysed at a dilution of 1:1,000. Rabbit anti-human IgG (whole molecule) − HRP antibody (1:4,000, Sigma-Aldrich, USA) was used as a secondary antibody and bound antibody was detected by incubation with 3,3′-diaminobenzidine substrate (Sigma, USA). As a negative control, we used the same group of sera from Argentina that was used in the ELISA assays (see above). By analogy to the reaction observed in mice (see Results and Discussion), in this study we considered that human sera reacting with three or four structural proteins were positive cases of exposure to TrV.

### Mathematical model to estimate *Triatoma virus* transmission rate

The number of potential infective contacts^b^ to which a susceptible person is exposed is one of the main factors in the transmission of a pathogen and thus a dominant factor in determining the prevalence of the disease produced by that pathogen. Provided that contact between an infected vector and a susceptible host generates a detectable effect (like replication of the parasite or an immune response against it), we can construct a general model based on the relationship between the prevalence of the infection, the number of potential contacts between vector and host, and the probability of the pathogen transmission. In the model developed below we assume that the population size of humans and vectors, the prevalence of the disease in humans, and the prevalence of the parasite in the vectors, are all constants in time and age-independent.

Let '*T* ' be the probability of transmission of a parasite from an infected vector to a susceptible (healthy) host, '*a*' the prevalence of the parasite in the vector's population, and '*N*' the total susceptible individuals in a given human population.

After the first potentially infective contact, the number of individuals infected with the parasite is *C*_*1*_, and given by:

1 $$ {C}_1=aTN $$

In a second contact, the number of new infected individuals is:

2 $$ \begin{array}{ll}{C}_2& =aT\left(N-{C}_1\right)=aT\left(N-aTN\right)\\ {}& =aTN\left(1-aT\right)\end{array} $$

In a third contact:

3 $$ \begin{array}{ll}{C}_3& =aT\left(N-C1-C2\right)\\ {}& =aT\left(N-aTN-aTN\left(1-aT\right)\right)\\ {}& =aTN{\left(1-aT\right)}^2\end{array} $$

And at the bite number *m*:

4 $$ \begin{array}{ll}{C}_m& =aT\left(N-C1-C2-\dots -Cm-1\right)\\ {}& =aTN{\left(1-aT\right)}^{m-1}\end{array} $$

Summing up the number of new infected individuals between the first to *m* contacts (*C*_tot_), the total fraction of infected individuals is:

5 $$ {C}_{tot}/N=1/N{\sum}_{j=1}^mCj=aT{\sum}_{j=1}^m{\left(1-aT\right)}^{j-1} $$

Equation 5 represents the fraction of the population that was infected after '***m***' potentially infective contacts with the vector, i.e., the prevalence.

Taking into account the property of geometric series:

6 $$ \frac{a}{1-x}={\displaystyle \sum_{p=0}^{\infty }}a{x}^p\mathrm{with}\ \left|x\right|<1 $$

Applying eq (6) to eq. 5 we arrive at the following expressions (see Additional file 1 for demonstration):

7 $$ m=\frac{ln\left(1-{P}_m\right)}{ln\left(1-a\cdotp T\right)} $$

8 $$ T=\frac{1}{a}\left(1-\sqrt[m]{1-{P}_m}\right) $$

Being *P*_*m*_ the prevalence after *m* potentially infective contacts, i.e., *P*_*m*_ = *C*_tot_/*N*.

### Estimation of the number of triatomine bites

In Chagas disease endemic areas, and considering only vectorial, transmission, the prevalence of the disease is directly related to the degree of vector exposure, which can be expressed as number of potentially infective bites. We can apply the mathematical model derived above to estimate the number of bites per person (*m*) affecting a population. To do so, the necessary data are the serological prevalence of *T. cruzi* (*C*_tot_/*N*), the *T. cruzi* prevalence in the insects (*a*_*Tc*_*)*, and the probability of parasite transmission to humans by an infected triatomine (*T*_*Tc*_*)*. The values of *C*_*tot*_*/N* and *a*_*Tc*_ can vary widely from one geographical point to another, and the literature contains abundant data for these two values that were obtained in specific locations. We used the value measured in Chaco, Argentina, with *C*_*tot*_*/N* = 27.81% and *a*_*Tc*_ = 0.301 [19]. The parameter *T*_*Tc*_ cannot be measured directly and depends on many factors, such as the triatomine species involved, the insect density, prevalence in the vector, etc. We used from the bibliography the *T*_*Tc*_ values estimated for *T. infestans* in Argentina: 0.000202 [4] and 0.00058 [3], and applied them in equation 7 (as *T = T*_*Tc*_) to estimate the number of bites or contacts between humans and triatomines in Chaco, Argentina.

### Estimation of Triatoma virus transmission to humans

If TrV enters the bloodstream of a susceptible host, the immune system will produce a response that can be detected by different assays, and allow distinguishing people who have been in contact with the virus from people who have not. Under these assumptions eq 8 can be used to compute the probability of TrV transmission (*T*_*TrV*_) to humans. TrV prevalence in insects (*a*_*TrV*_) can also be estimated. Given those pieces of data it is possible to estimate the number of bites (*m*) from eq. 7, and estimate the probability *T*_*TrV*_. The prevalence of TrV was based on the average value estimated in Chaco’s neighbouring provinces of Santa Fe and Santiago del Estero [13], resulting in *a*_*TrV*_ = 0.127.

## Results and discussion

### Antibodies against structural proteins of *Triatoma virus*

**Figure 1:**
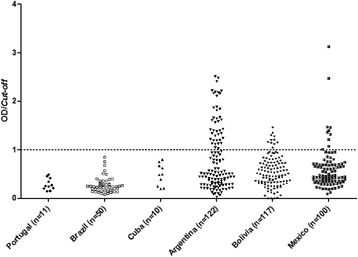
**ELISA assay 1 analysis of human sera.** The results of the IgG antibodies against TrV structural proteins were analysed at an absorbance of 490 nm. The cut-off value was the mean of the optical density (OD) plus three times the standard deviation, so sera samples with OD/cut-off values greater than 1.1 were considered positive; sera samples with OD/cut-off values 0.9 < OD/cut-off < 1.1 were considered indeterminate, and sera samples with values of OD/cut-off < 0.9 were considered negative.

The samples obtained from Brazil, Cuba and Portugal displayed relatively weak serological reactivity, which suggests that people might have never been exposed to TrV (Figure 1, Table 1). Sera from Cuba were obtained from people with no history of Chagas disease, so the results are consistent, since the probability of these people having been in contact with triatomines or TrV are too low. On the other hand, the sera from Portugal are imported case of Chagas disease, mainly from Brazil. So, the results from Portugal and Brazil are very consistent, since to date TrV was found in only one insectary from Brazil, which may indicate the low prevalence of this virus in natural population of triatomines from Brazil.

33.6% (n = 41) of sera analysed from Argentina, 9.4% (n = 11) from Bolivia and 9% (n = 9) from Mexico showed high levels of anti-TrV antibodies (Figure 1), suggesting that individuals from those countries had been exposed to this virus (Table 1). From the positive Argentinean sera samples, five (12.2%) corresponded to healthy individuals and the rest to Chagas patients. All positive TrV sera samples from Bolivia and from Mexico corresponded to patients with Chagas disease.

**Figure 2:**
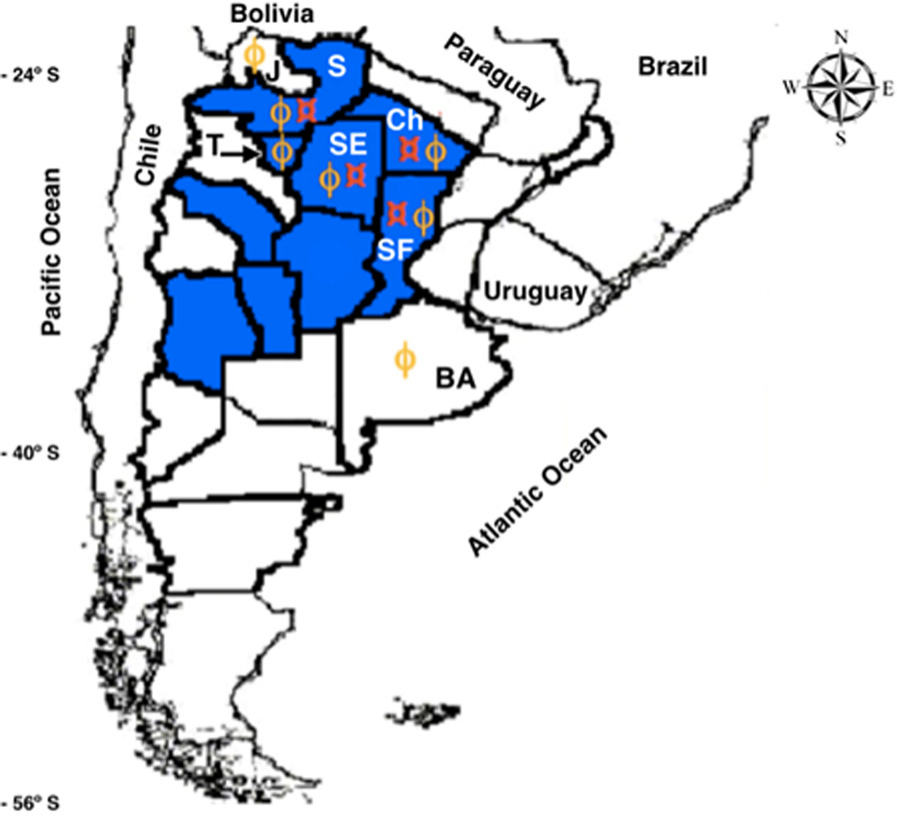
**Geographical distribution of TrV prevalence in triatomines, and provinces of origin of the sera from Argentina.** TrV was found in a region that covers nine provinces (colored in blue) [20]. Red currency signs (**¤**) identify the four provinces where the 36 sera sample with three positive immunological reactions against TrV to TrV: Chaco (Ch), Santa Fe (SF), Santiago del Estero (SE) and Salta (S). The remaining provinces with negative sera for TrV (86) were Buenos Aires (BA), Jujuy (J), and Tucumán (T). Yellow Cyrillic Ef (ϕ) identifies provinces origin of sera analysed.

Because the distribution of TrV is widespread in the northern provinces of Argentina (Figure 2), it is not surprising that sera from Bolivian patients were positive for the virus.

We would have not expected people from Mexico to have been in contact with TrV, but although there are no reports of TrV in triatomines from North America, the triatomine species *Meccus longipennis* from a Brazilian insectary was positive for TrV, and the colony was originally from Mexico [12]. The occurrence of TrV positive human sera samples from this country suggests that the virus may have spread throughout the Americas.

### Confirmation of exposure to *Triatoma virus* in inhabitants from Argentina

When sera from mice inoculated with TrV were tested by immunoblotting (WB) assays, three or four bands were detected (data not shown). These bands corresponded to VP0, VP3, VP1, and VP2. Therefore this assay, if positive, can be used as confirmatory evidence of contact with TrV.

**Figure 3:**
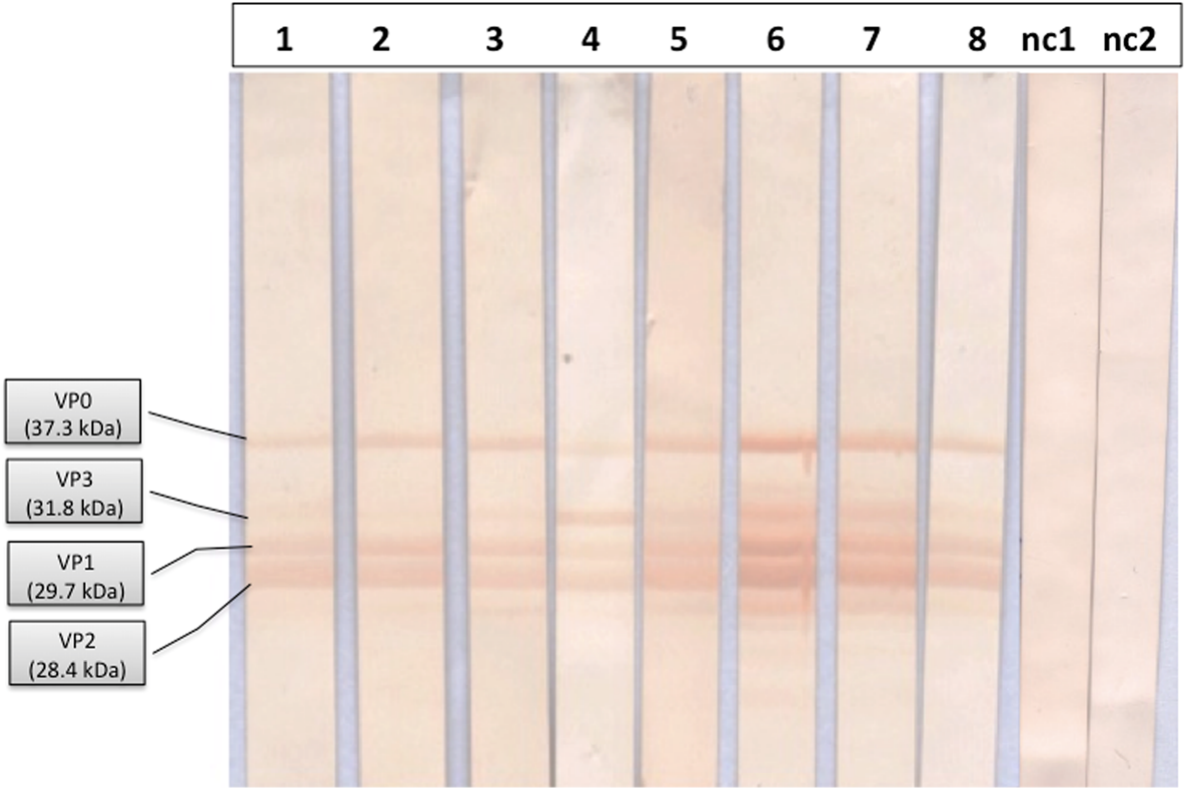
**Immunoblotting analysis against the structural proteins of** ***Triatoma virus*** **.** Each strip was loaded with 5 μg of capsid protein from TrV (e-TrV). Lines 1–8 show reactive sera of eight selected samples. Out of a total of 50 analysed samples, 36 resulted positive, i.e., showed reactivity against three or four VPs; Iinesnc1 and nc2 correspond to sera of individuals from *Triatoma virus*-free provinces of Argentina.

Because TrV was found only in populations of triatomines from Argentina, it is likely that people in Chagas endemic areas from this country might have been exposed to the virus. From the immunoblotting analysis of the fifty sera samples from Argentina identified as SeraA^+^, 36 sera samples (72%) were positive to TrV (Figure 3, Table 1). These 36 sera samples from Argentinean individuals can be considered confirmed cases of exposure to TrV; of those 36 cases five (13.9%) were negative to *T. cruzi.* Interestingly, all these people inhabit provinces reported as endemic for TrV [13] (Figure 2).

### Triatoma virus is unable to replicate in human hosts

Viruses with a genome composed of -ssRNA and dsRNA enclose their own RdRp within the viral capsid [21]. In contrast to these viruses, TrV is a + ssRNA virus and does not contain this polymerase within the viral capsid. This implies that to have effective replication within a host cell, TrV needs to promote the synthesis of all non-structural and structural protein precursors. So, only in case of active viral replication, the host immune system can produce antibodies that recognize both the structural and non-structural viral proteins. When a reaction to both groups of proteins is determined, this not only indicates that the host was exposed to the virus but also that the pathogen was able to replicate by expressing its own proteins through the use of the host cell machinery. On the contrary, if only antibodies against structural proteins are found, this implies that exposure to the virus was passive [22].

**Figure 4:**
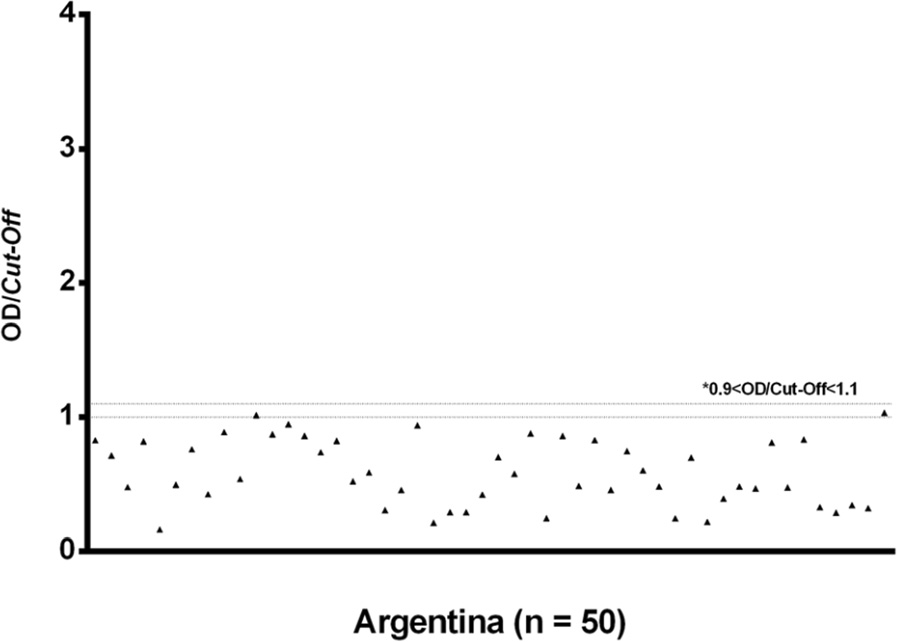
**ELISA assay 2 analyses against RdRp of TrV.** RdRp is a non-structural protein of TrV, and negativity to this protein indicates that the virus does not replicate in the infected host. Sera samples with a ratio of OD/cut-off smaller than 1.1 were considered negative.

To differentiate passive exposure to TrV from active viral replication, we analysed the subgroup SeraA^+^ samples by an ELISA against recombinant RdRp from TrV (ELISA assay 2). Our results showed that there was no serological reactivity against recombinant RdRp from TrV (Figure 4, Table 1).

**Figure 5:**
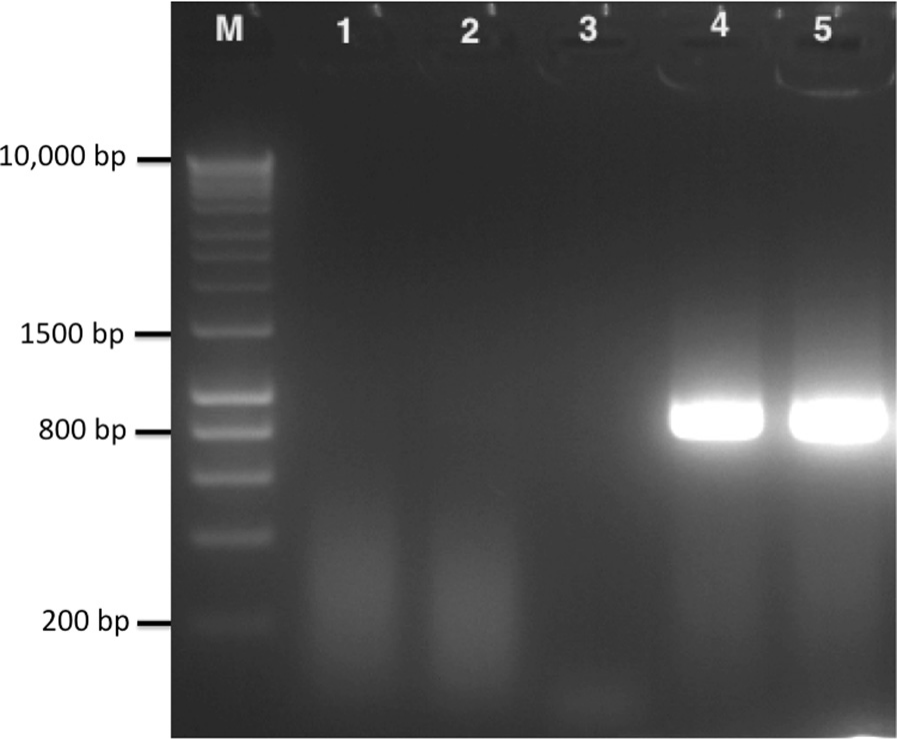
**Molecular diagnosis of TrV in human sera by RT-PCR.** Lane 1: human sera from a non-endemic country; Lane 2: Pool of negative sera used in immunochemical assays (Western blot); Lane 3: Pool of positive sera analysed by immunochemical assays (Western blot); Lane 4: 5 μl of pure TrV diluted in serum; Lane 5: 5 μl of pure TrV diluted in water.

Reverse Transcriptase-PCR (RT-PCR) is widely used in molecular diagnosis of RNA viruses. In this study, we used RT-PCR for molecular diagnosis of TrV in the SeraA^+^ subgroup samples, and there were no positive results (Figure 5, Table 1). These results, together with the ELISA assay 2, indicate that TrV is unable to replicate in the human host, as it was observed in sera samples from mice experimentally inoculated with TrV [18].

### The probability of *Triatoma virus* transmission to humans is greater than that of *Trypanosoma cruzi*

**Figure 6:**
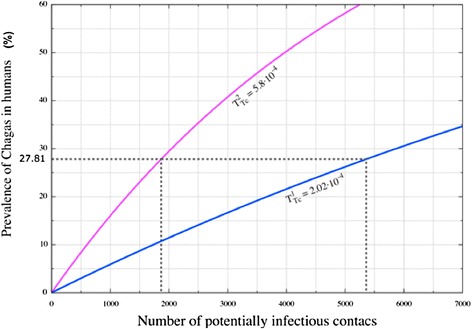
**Estimation of the number of potential infective contacts for Chagas patients in Chaco (Argentina).** Predicted prevalence to *T. cruzi* in humans computed from our mathematical model of infection (eq. 5) using a *T. cruzi* prevalence value in triatomines of *a*_*Tc*_ = 0.301,from a specific population of Chaco, Argentina [18]. Two values of the transmission rate parameter *T*_*Tc*_ = 0.000202 [4] and *T*_*Tc*_ = 0.00058 [3] were used. From eq. 7 we calculated, for a given prevalence of *T. cruzi* in humans of 27.81% (from the same population of Chaco, Argentina [18]), the number of potentially infective bites that the human population would be exposed to, resulting in 1,866 and 5,359 bites (magenta and blue curves, respectively).

The calculation of human *T. cruzi* prevalence for Chaco, Argentina, using the mathematical model under the assumption of a prevalence of *T. cruzi* in triatomines of aTc = 0.301 [19], and as a function of various numbers of potentially infective contacts, are shown in Figure 6, for two values for the *T. cruzi* transmission rate (*T*^*1*^*Tc* = 0.00053, and *T*^*2*^*Tc* = 0.000202) [3,4]. Using eq. 7, and the *T. cruzi* field prevalence value of 27.81% for that region, we estimated the number of potentially infective bites that people would have been exposed to: 1,886 (= m_1_) and 5,359 (= m_2_), for the two *TTc* values used; these two estimates are also shown in Figure 6. Thus, using eq. 8 the two transmission probabilities of TrV to humans are *T*^*1*^*TrV* =0.001709 for m = m_1_, and *T*^*2*^*TrV* = 0.0006016 for m = m_2_. Both results indicate that the probability of TrV transmission to humans is about three times greater than the transmission rate of *T. cruzi* to humans (*T*^*1*^*TrV* /*T*^*1*^*Tc* = 3.22 and *T*^*2*^*TrV* /*T*^*2*^*Tc* = 2.97). This estimation, of course, is specifically for the population of Chaco and cannot be generalized to other regions.

As the sera samples were randomly selected, they should be representative of the true prevalence of people seropositive to TrV. This value is approximately 30%, whereas the average prevalence of the virus in triatomines is approximately 10%; this relationship, and the surprisingly high percentage of people positive to TrV we found in Argentina has to be explained. One possibility is the possible difference in the mechanism of entrance to the human bloodstream between TrV *T. cruzi*. Although both pathogens infect by ingestion of contaminated food or by contaminated feces entering a skin wound or the conjunctiva, *T. cruzi* is destroyed in dry triatomine feces and high temperatures [23], while TrV can remain viable at room temperature for many months or years in dry triatomine feces [14]. This resistance to adverse climatic conditions (high ambient temperature and low relative humidity) could be a factor to increase its transmission probability to humans. For instance, human contact with TrV may occur by breathing of infected feces dust (Additional file 2: Figure S1), although the confirmation of this route of infection remains to be shown.

Our estimation of the transmission rate of TrV from an infected vector to a susceptible human host is based on the assumption that in an individual insect the co-infection of *T. cruzi* and TrV is possible; however, an evaluation of this possibility has never been investigated, and goes beyond the goal of this work; as it is a critical hypothesis to the analysis of the transmission of both TrV and T. cruzi, it justifies more research.

## Conclusions

We presented the first experimental evidence of antibodies against TrV structural proteins in human sera. Because we aimed to analyse people more likely to have viral exposure, and because TrV is released with insect feces, the majority of the sera analysed in this study came from Chagas disease patients. Our findings contrast with a previous study that failed to demonstrate viral exposure in patients with Chagas disease from Argentina [10]. Nevertheless, our conclusion that TrV does not replicate in humans is in agreement with previous observations of other dicistroviruses that, in spite of having been in contact with hens, horses, cows, rabbits and sheep, did not replicate [22].

To date there is no effective vaccine for Chagas disease prevention, so vector control has been the main mechanism to decrease *T. cruzi* transmission [1]. Because knowledge of the biology of several members of the *Dicistroviridae* family has increased, the possibility of using viruses with RNA genomes as biological control agents for insects looks promising [24]. Our results indicate that TrV seems unable to replicate in human hosts, and this is consistent with our recent observation that this virus is also unable to replicate in mice [18]. These findings suggest that TrV (and most likely all dicistroviruses) do not pose any risk to humans nor wild and domestic animals, leaving open the possibility of using this virus as biopesticide to control the vectors of Chagas disease.

## Endnotes

^a^Previous studies have found virus-like particles in triatomines from Brazil [20,25]. However, these particles were extracted from insects showing no sign of disease and were not further characterized aside from electron microscopy and RNA analysis. The estimated diameter of those particles was 38-40 nm, larger than the 30 nm of TrV.

^b^An ‘infective contact’ can be a bite of the insect vector, ingestion of contaminated food, incorporation of the parasite through the mucosa, etc.

## Additional files

Additional file 1:
**Analytical proof of eq.** **7** **and eq.** **8** **from eq.** **5** **using a geometric series approximation.**
Additional file 2: Figure S1.
*T. infestans* inhabits houses and domestic animal shelters. The insect's “nests” are generally in cracks of the plastering or between the joints of the roof with the walls. After feeding on their hosts, the insects return back to their “nest” and defecate on their way. The feces containing TrV are exposed to hot and dry air favoring ambient contamination, resulting in a potential source of TrV particles. Picture taken in a hen shelter in a rural area of Camiri, Bolivia (October 2012).
