# Supplementary material for: Seroprevalence of Triatoma virus (Dicistroviridae: Cripaviridae) antibodies in Chagas disease patients
Source: Parasit Vectors. 2015 Jan 17;8:29. doi: 10.1186/s13071-015-0632-9 (PMC4351825; doi:10.1186/s13071-015-0632-9)
Supplement: Additional file 1: — Analytical proof of eq. 7 and eq. 8 from eq. 5 using a geometric series approximation. [file 13071_2015_632_MOESM1_ESM.pdf]

Annex

Analytical proof of eq. 7 and eq. 8 from eq. 5 using a geometric series approximation

Eq. 5 expressed the proportion of the accumulated new cases (C_{to}) after m potentially infective contacts as:

$$P_m = C\text{tot}/N \ = aT \sum_{n=1}^{m} (1 - aT)^{n-1} \qquad (5)$$

Where T is the transmission rate of a pathogen to a susceptible host after contact with one infected vector, and a is the prevalence of infection of vectors for that same pathogen.

The summation can be extended to infinity:

$$\sum_{n=1}^{\infty} (1 - aT)^{n-1} = \sum_{n=1}^{m} (1 - aT)^{n-1} + \sum_{n=m+1}^{\infty} (1 - aT)^{n-1} \qquad (5')$$

Changing the index $\qquad p = n - 1$

$$\sum_{p=0}^{\infty} (1 - aT)^{p} = \sum_{p=0}^{m-1} (1 - aT)^{p} + \sum_{p=m}^{\infty} (1 - aT)^{p} \qquad (5'')$$

We can use the property of a geometric series:

$$\frac{1}{1 - x} = \sum_{p=0}^{\infty} x^p \quad \text{for} \quad |x| < 1 \qquad (6)$$

And as $|1 - aT| < 1$, we can replace $x = 1 - aT$, so that eq. (6) becomes

$$\sum_{p=0}^{\infty} (1 - aT)^{p} = \frac{1}{aT}$$

The second term of the right of eq. 5'' can be transformed by an index change by re-defining $p = q + m$ so:

$$\sum_{q=0}^{\infty} (1 - aT)^{q+m} = (1 - aT)^{m} \cdot \frac{1}{aT}$$

Thus, eq. 5 becomes:

$$P_m = C\text{tot}/N = aT \sum_{p=0}^{m-1} (1 - aT)^p = 1 - (1 - aT)^m$$

Figure S1. *T. infestans* inhabits houses and domestic animal shelters. The insect's “nests” are generally in cracks of the plastering or between the joints of the roof with the walls. After feeding on their hosts, the insects return back to their “nest” and defecate on their way. The feces containing TrV are exposed to hot and dry air favoring ambient contamination, resulting in a potential source of TrV particles. Picture taken in a hen shelter in a rural area of Camiri, Bolivia (October 2012).
