# Supplementary figures and images for: Seroprevalence of Triatoma virus (Dicistroviridae: Cripaviridae) antibodies in Chagas disease patients
Source: Parasit Vectors. 2015 Jan 17;8:29. doi: 10.1186/s13071-015-0632-9 (PMC4351825; doi:10.1186/s13071-015-0632-9)

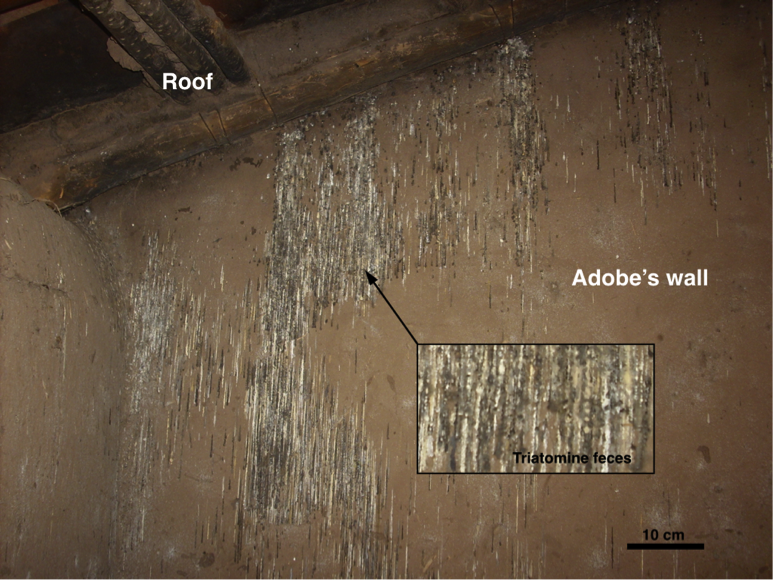

Supplement: Additional file 2: Figure S1. — T. infestans inhabits houses and domestic animal shelters. The insect's “nests” are generally in cracks of the plastering or between the joints of the roof with the walls. After feeding on their hosts, the insects return back to their “nest” and defecate on their way. The feces containing TrV are exposed to hot and dry air favoring ambient contamination, resulting in a potential source of TrV particles. Picture taken in a hen shelter in a rural area of Camiri, Bolivia (October 2012). [file 13071_2015_632_MOESM2_ESM.png]
